# Generation of MicroRNA-34 Sponges and Tough Decoys for the Heart: Developments and Challenges

**DOI:** 10.3389/fphar.2018.01090

**Published:** 2018-09-21

**Authors:** Bianca C. Bernardo, Paul Gregorevic, Rebecca H. Ritchie, Julie R. McMullen

**Affiliations:** ^1^Baker Heart and Diabetes Institute, Melbourne, VIC, Australia; ^2^Department of Paediatrics, The University of Melbourne, Melbourne, VIC, Australia; ^3^Department of Diabetes, Central Clinical School, Monash University, Clayton, VIC, Australia; ^4^Department of Physiology, Centre for Muscle Research, The University of Melbourne, Melbourne, VIC, Australia; ^5^Department of Pharmacology and Therapeutics, The University of Melbourne, Melbourne, VIC, Australia; ^6^Department of Medicine, Monash University, Clayton, VIC, Australia; ^7^Department of Physiology, Monash University, Clayton, VIC, Australia; ^8^Department of Physiology, Anatomy, and Microbiology, La Trobe University, Melbourne, VIC, Australia

**Keywords:** microRNAs, heart failure, tough decoy, microRNA sponge, antisense oligonucleotides

## Abstract

Heart failure (HF) is a debilitating and deadly chronic disease, with almost 50% of patients with HF dying within 5 years of diagnosis. With limited effective therapies to treat or cure HF, new therapies are greatly needed. microRNAs (miRNAs) are small non-coding RNA molecules that are powerful regulators of gene expression and play a key role in almost every biological process. Disruptions in miRNA gene expression has been functionally linked to numerous diseases, including cardiovascular disease. Molecular tools for manipulating miRNA activity have been developed, and there is evidence from preclinical studies demonstrating the potential of miRNAs to be therapeutic targets for cardiovascular disease. For clinical application, miRNA sponges and tough decoys have been developed for more stable suppression and targeted delivery of the miRNA of choice. The aim of this study was to generate miRNA sponges and tough decoys to target miR-34 in the mouse heart. We present data to show that using both approaches we were unable to get significant knockdown of miR-34 or regulate miR-34 target genes in the heart *in vivo*. We also review recent applications of this method in the heart and discuss further considerations for optimisation in construct design and testing, and the obstacles to be overcome before they enter the clinic.

## Introduction

Heart failure (HF), the clinical manifestation of numerous forms of cardiovascular disease, is a devastating disorder and a significant global health problem (Braunwald, 2015; Mozaffarian et al., 2015). There are very limited therapies or procedures to treat or cure HF, thus novel therapeutics that target the underlying causes and not only the symptoms of the disease need to be developed. microRNAs (miRNAs) have come into focus as potential novel therapeutics for the treatment of cardiovascular disease. In their mature form, miRNAs are small (∼22 nucleotides), endogenous non-coding RNA molecules that can regulate gene expression (Bernardo et al., 2012a, 2015; Hata, 2013). Binding of miRNAs to the 3′ untranslated region of target messenger RNAs (mRNAs) causes mRNA degradation or translational repression. miRNAs can target hundreds of mRNAs, and thus regulate several molecular pathways, rendering them powerful regulators compared to conventional mono-target therapeutics (Bernardo et al., 2012a, 2015; Hata, 2013). miRNAs have been shown to play a key role in cardiovascular structure and function (Chen et al., 2008) and cardiac remodeling in response to a pathological stress (Care et al., 2007; Callis et al., 2009). Further, perturbed miRNA expression has been linked to cardiovascular disease (Small and Olson, 2011; Nouraee and Mowla, 2015), suggesting miRNAs as a new class of therapeutic targets. An exciting development in the miRNA field is the efficient manipulation of miRNA activity using molecular tools to normalize gene expression in a diseased state. The most successful molecular tools to date are antisense oligonucleotides (ASOs, which act as miRNA inhibitors), which have already entered clinical trials in patients with hepatitis C (Janssen et al., 2013), cancer, type 2 diabetes, and non-alcoholic fatty liver diseases (Chakraborty et al., 2017; Rupaimoole and Slack, 2017). Conversely, strategies have been developed to reintroduce depleted miRNAs into diseased cells, either using oligonucleotide-based double-stranded miRNA mimics, or by the use of viral vectors to drive overexpression of the miRNA of interest. Montgomery et al. (2014) developed a functional synthetic RNA duplex that was able to successfully restore miR-29 to treat fibrotic disorders *in vivo*, whilst another group used a viral vector approach to restore miRNA-29 activity in a mouse model of Duchenne muscular dystrophy (Heller et al., 2017). Despite this progress, *in vivo* delivery of miRNA mimics remains a challenge. Improvements in delivery systems, dosing schedule, cellular uptake and *in vivo* stability will need to be addressed. In this perspective, we will focus on strategies that inhibit miRNAs *in vivo*.

Given miRNAs are ubiquitously expressed, and miRNA inhibitors are taken up by numerous organs upon systemic delivery (Obad et al., 2011), for miRNA-based therapies to enter the clinic for the treatment of cardiovascular disease, tissue-specific regulation of miRNAs is highly desirable. Recently, several methods to increase miRNA target specificity have been developed using vector-based strategies. There are, however, very few descriptions of studies using this technology in the literature for the heart (Xie et al., 2012; Jeong et al., 2018; Nie et al., 2018).

## Targeting miRnas for Long Term and Efficient Inhibition in the Heart

### Regulation of miRNAs With Pharmacological Inhibitors

The most common approach to inhibit miRNA in the heart is to employ chemically modified ASOs. The most commonly used ASOs include antagomiRs (which target single miRNAs conjugated with cholesterol), antimiRs and tiny antimiRs (that target single miRNAs or miRNA families, respectively) often with locked nucleic acid (LNA) modifications (see review Bernardo et al., 2015). There has been a plethora of preclinical studies demonstrating the therapeutic potential of inhibiting miRNAs using ASOs (see reviews: Small and Olson, 2011; Hata, 2013; Ooi et al., 2014; Bernardo et al., 2015). For example, inhibition of phosphoinositide 3-kinase (p110α) (PI3K)-regulated miRNAs (miR-34a, miR-34 family, miR-652, and miR-154) was associated with favorable cardiac function, decreased fibrosis and/or increased angiogenesis in mouse models of pressure overload and/or myocardial infarction (MI) (Bernardo et al., 2012b, 2014a,b, 2016a). Targeting the miR-15 family and the miR-302-367 cluster has been shown to regulate cardiomyocyte proliferation and improved cardiac function after MI (Porrello et al., 2011; Tian et al., 2015). Further, miR-208 has been shown to regulate cardiac contractility and energy metabolism (Montgomery et al., 2011; Grueter et al., 2012).

### Regulation of miRNAs With Viral Vectors

MicroRNAs have been successfully regulated in the heart using an adenoviral approach [e.g., miR-24 and miR-133; (Care et al., 2007; Meloni et al., 2013)]. More recent approaches include the development of AAV combined with miRNA sponges and tough decoys; first developed in 2007 and 2009, respectively (Ebert et al., 2007; Haraguchi et al., 2009). miRNA sponges are plasmid constructs that contain multiple high affinity miRNA antisense binding sites. These transcripts can efficiently sequester specific miRNAs, preventing their binding to endogenous target genes. The experimental application of miRNA sponge technology is a valuable tool for miRNA loss-of-function studies *in vivo*. This technology has been successfully used in laboratories around the world to regulate miRNAs specifically in photoreceptor cells in the eye (Krol et al., 2010) and to regulate miRNAs in skeletal muscle (Winbanks et al., 2013). On commencing our own studies, there were limited reports describing AAV sponges/tough decoys in the heart, where one study reported increased expression of miRNA targets genes in the heart, but the expression of the miRNA was not directly measured (Xie et al., 2012).

## The Development of miRna Sponges and Tough Decoys Targeting miR-34 Family or miR-34a

In a previous study, we identified miRNAs increased in the heart in a cardiac disease setting (MI) and decreased in a protected setting (via transgenic expression of the cardioprotective protein PI3K) (6). This unique approach of identifying miRNAs by our laboratory led to subsequent demonstration that the inhibition of PI3K-regulated miRNAs (using miRNA inhibitors) was associated with reduced pathology and more favorable left ventricular systolic function in mouse models of cardiac disease (Bernardo et al., 2012b, 2014a,b, 2016a,b). Although we (Bernardo et al., 2012b, 2014a, 2016b), and others (Boon et al., 2013; Yang et al., 2015), were able to show that inhibition of miR-34a or the miR-34 family is protective in the hearts of mice following a cardiac insult (but inhibition had no phenotypic effect in control mice), chronic inhibition of miR-34 may not be ideal because of its ability to drive tumorigenesis (Misso et al., 2014). Thus, these concerns warranted development of a targeted therapeutic approach, to directly inhibit miR-34 in the heart. The aim of this study was to generate miR-34 sponges and a miR-34 tough decoy, and assess the degree of knockdown of miR-34/miR-34a in the normal healthy adult mouse heart. To do this, we used vector-based strategies that contain multiple high affinity miRNA antisense binding sites, which can sequester specific miRNAs, preventing their binding to endogenous target genes, thereby acting as “decoys” or “sponges” (Bak et al., 2013; Bak and Mikkelsen, 2014; Xie et al., 2015). We hypothesized that expression of miR-34/miR-34a would be significantly reduced in the heart if the sponges and tough decoy were to work similar to the antisense oligonucleotides. However, the sponge or tough decoy would have no phenotypic effect on the normal adult mouse heart, as previously described with miRNA inhibitors (Bernardo et al., 2012b). If significant miR-34 knockdown was achieved, the next goal was to test the miR-34 sponges/tough decoy in HF mouse models in which we would expect significant miR-34 knockdown, together with improved cardiac outcomes (Bernardo et al., 2012b).

**FIGURE 1:**
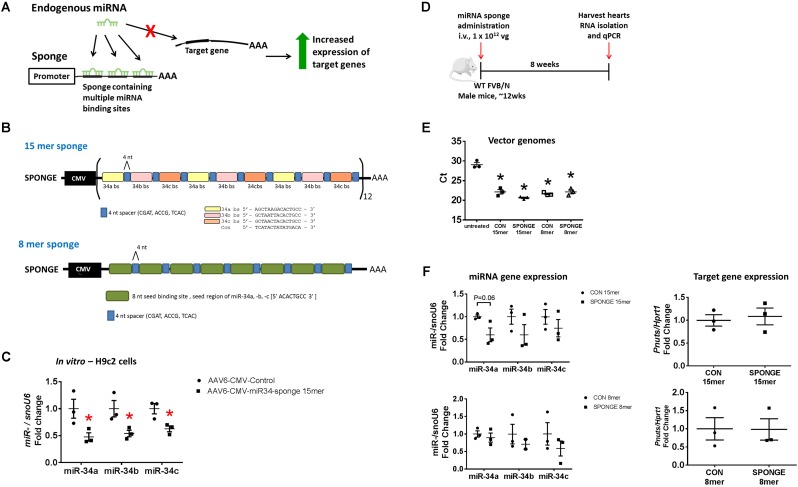
Design and testing of a miR-34 sponge. **(A)** A construct containing multiple miRNA binding sites act as a competitive inhibitor and “soaks up” endogenous miRNA preventing binding to target genes, resulting in increased expression of target genes. **(B)** A schematic showing the design of the miR-34a 15mer and 8mer sponge. The 15mer sponge contains four tandem repeats of the binding sites for miR-34a (yellow), miR-34b (pink), and miR-34c (orange), four nucleotide spacers (blue), under the control of the CMV promoter. The 8mer sponge contains eight tandem repeats of the miR-34 seed region (green), four nucleotide spacers (blue), under the control of the CMV promoter. **(C)** miR-34a, -b, and -c gene expression in H9c2 cells transfected with AAV6-CMV control or AAV6-CMV-miR-34 15mer sponge. Data shown as mean ± SEM. Un-paired *t*-test. ^∗^*P* < 0.05. *N* = 3/group. **(D)** Experimental timeline. **(E)** Detection of vector genomes in untreated, AAV6:CON and AAV6:sponge (15mer and 8mer) treated hearts. Lower Ct (cycle threshold) indicates higher expression. *N* = 3/group. Data mean ± SEM. One way ANOVA with Fisher’s *post hoc* test. ^∗^*P* < 0.05 vs. untreated. **(F)** Expression of miR-34a, miR-34b, and miR-34c (left) and *Pnuts* (right) by qPCR in control and miR-34 sponge 15mer and 8mer treated hearts. Data mean ± SEM. *N* = 3/group (except *N* = 2 for miR-34b in 8mer sponge). Abbreviations: AAA, poly A tail; bs, binding site; i.v., intravenous; vg, vector genomes.

We produced two versions of a miR-34 sponge, both based on the design by Gentner et al. (2009) and Kluiver et al. (2012b). miRNA sponges contain repeated miRNA-binding sites which act as competitive inhibitors for miRNA binding (**Figure 1A**; Bernardo et al., 2015). Sponges are designed to include 4–12 multiple binding sites to sequester miRNAs, either fully or partially complementary to the miRNA target of interest (**Figure 1A**; Bernardo et al., 2015). It has been reported that increasing the number of binding sites in a single sponge enhances sponge activity, although saturation can occur and the risk of RNA degradation and genetic recombination increases (Ebert et al., 2007). In our sponge design, we included a short 4 nucleotide (nt) sequence modification, termed “spacers,” to separate each binding site to optimize the binding of miRNA to every possible binding site and reduce the chance of formation of RNA secondary structures (**Figure 1B**). To target the miR-34a family, we used the “minigene” approach described by Kluiver et al. (2012a). Our construct consisted of four tandem repeats of either the miR-34a, miR-34b, and miR-34c binding sites (binding sites are 15-nt long, termed “15mer”), or eight tandem repeats of the miR-34 seed region (seed region is 8-nt long, termed “8mer”). Each binding site was separated by a 4-nt spacer (**Figure 1B**). For the control, the same construct was synthesized but contained a scrambled sequence (either 15-nt or 8-nt) in place of a miRNA binding site. These scrambled sequences have previously been used *in vivo*, eliciting no effect (Bernardo et al., 2012b, 2014a, 2016a). We expressed the miR-34 sponges in adeno-associated virus vector 6 (AAV6) under the cytomegalovirus (CMV) promoter. We previously showed that the combination of the AAV6 serotype and promoter preferentially and efficiently transduced cardiac muscle, and to a lesser extent, skeletal muscle, and no expression was detected in lung, kidney, or spleen. (Gregorevic et al., 2004; Weeks et al., 2012; Bernardo et al., 2018). Therefore, we do not expect our sponges or tough decoys within the AAV6-CMV cassette to have a functional effect in other tissues.

We first tested the AAV6-CMV-miR-34 15mer sponge *in vitro*. Following transfection of H9c2 cells (a cardiomyoblast cell line) with AAV6-CMV-control or -miR-34 15mer sponges, there was ∼40–50% knockdown of miR-34a, -b, and -c gene expression in treated cells compared to control cells (**Figure 1C**). As the 8mer was of the same design (but only contained the seed region) we proceeded to test these sponges *in vivo*. We initially performed an experiment where we injected adult wildtype mice with two different doses of AAV6-miR-34 15mer sponge. The first dose (2 × 10^11^ vector genomes [vg]) was based on our previous publications (Weeks et al., 2012; Prakoso et al., 2017; Bernardo et al., 2018) where we had observed effective transduction in the heart with AAV6 carrying mRNAs. The second, higher dose (1 × 10^12^ vg) was chosen as this dose had been used by others (Xie et al., 2012). As AAV transduction of murine myocardium typically takes 11 days (Gregorevic et al., 2004) we harvested hearts 3 weeks post AAV6-CMV-miR-34 15mer sponge administration for molecular analysis. We performed qPCR for miR-34a and found that after 3 weeks, there was no knockdown in the heart (*N* = 3/group, data not shown). We then proceeded to a longer time point.

To test the effectiveness of miR-34 sponge designs in sequestering miR-34 family members in the heart, we administered one single dose of miR-34 sponge intravenously to wildtype adult male mice, and collected the heart for molecular analysis 8 weeks later (**Figure 1D**). We first examined whether we could detect vector genomes in the heart. Using primers specific to the CMV promoter, there was an ∼6-cycle difference (i.e., 2^6^ change) in vector genomes between the hearts of untreated mice and hearts of mice treated with both AAV6-control (15mer and 8mer) or AAV6-miR-34a-sponges (15mer and 8mer) (**Figure 1E**), indicating that the AAV6-sponges and relevant controls were taken up by the heart. We next examined the gene expression of miR-34 family members in the heart and observed that administration of the AAV6-miR-34-sponge 15mer showed a tendency for decreased miR-34a expression (**Figure 1F**, *P* = 0.06) compared to control, but the expression of miR-34b and miR-34c was not significantly different. The AAV6-miR-34 sponge 8mer design did not inhibit miR-34 family members in the heart as we would have expected (**Figure 1F**). It has previously been recommended that direct measurements of the targeted miRNA should be accompanied by assessment of functional effects after miRNA antagonism (Stenvang et al., 2012). Hence we next assessed the de-repression of a direct and validated target gene of miR-34a, protein phosphatase 1 regulatory subunit 10 (also known as *Pnuts*) (Boon et al., 2013), by real-time qPCR. Following administration of miR-34 15mer or 8mer sponges, the expression of *Pnuts* was not significantly different to that of control treated hearts (**Figure 1F**). Thus, it appears that AAV6-miR-34 sponges were not able to inhibit miR-34 family members in the heart, or to de-repress miR-34 target genes.

**FIGURE 2:**
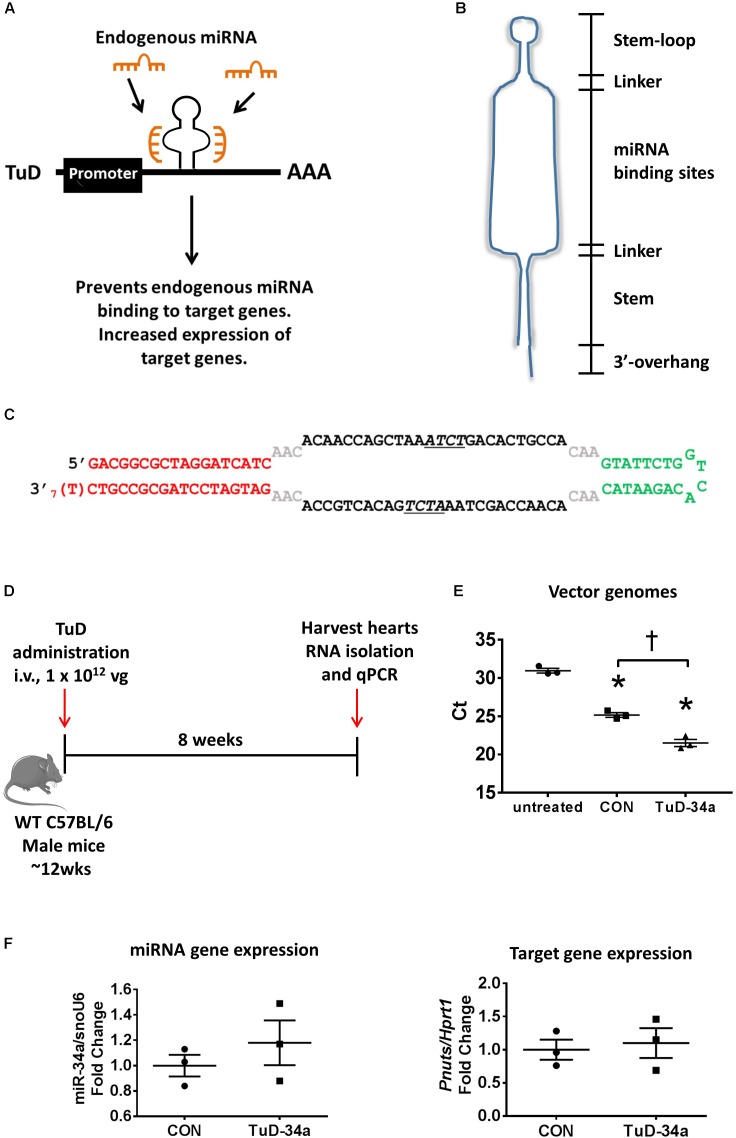
Design and testing of a tough decoy (TuD) targeting miR-34a. **(A)** A schematic of the TuD miRNA expression cassette design. The TuD acts as a competitive inhibitor and will bind endogenous miRNAs preventing them binding to target genes, resulting in increased expression of target genes. **(B)** TuD design. **(C)** Sequence of the TuD-miR-34a. **(D)** Experimental timeline. **(E)** Detection of vector genomes in untreated, control and TuD-34a treated hearts. Lower Ct (cycle threshold) indicates higher expression. *N* = 3/group. Data mean ± SEM. One way ANOVA with Fisher’s *post hoc* test. ^∗^*P* < 0.05 vs. untreated. ^†^*P* < 0.05. **(F)** Expression of miR-34a (left) and *Pnuts* (right) by qPCR in control and TuD-34a treated hearts. Data mean ± SEM. *N* = 3/group. Abbreviations: AAA, poly A tail; i.v., intravenous; TuD, tough decoy; vg, vector genomes.

To achieve greater knockdown, we next generated a “Tough Decoy” (TuD), as studies targeting other miRNAs were reported that the TuDs were more potent than miRNA sponges (Xie et al., 2012; Hollensen et al., 2013). Based on the design published by Xie et al. (2012), we generated a miR-34a TuD (**Figure 2A**). The TuD is a ∼60-base pair (bp) long hairpin-shaped RNA with an internal loop exposing two miRNA binding sites to support increased miRNA inhibition (**Figure 2B**). The miRNA-binding sites are complementary to the miRNA of interest (in this case, miR-34a) and acts as a competitive inhibitor preventing endogenous miRNA binding to target mRNAs (**Figure 2A**). Specifically, the TuD is composed of four elements: an 18-bp long stem, two miRNA-binding sites, a 26-nt long stem-loop structure connecting the miRNA binding sites and four 3-nt long linkers joining the two miRNA-binding sites with the stem-loop and the stem, respectively (**Figures 2B,C**). In each of the miRNA binding sites, a 4-nt long bulge is introduced between nucleotides 10 and 11 from the 5′ end of the miRNA to avoid perfect-base pairings with miRNAs (**Figures 2B,C**). This bulge prevents cleavage of the miRNA-binding site. We expressed the miR-34a TuD in the AAV6 vector with a CMV promoter. To test the effect of the TuD-miR-34a in sequestering miR-34a in the heart, we administered one single dose of TuD-miR-34 or AAV6-control intravenously to wildtype adult male mice and collected the heart for molecular analysis 8 weeks later (**Figure 2D**). Similar to our sponge experiment, we first confirmed that our TuD was taken up by the heart, as we could detect vector genomes in the hearts of mice treated with the AAV6-CON and TuD-34a compared to untreated hearts (**Figure 2E**). Gene expression analysis of miR-34a did not change (**Figure 2F**). Similarly, the expression of *Pnuts* was unchanged (**Figure 2F**). Thus, we were unable to achieve significant inhibition of miR-34a and miR-34 family members in the heart using these targeted approaches, when compared to our previous studies using LNA inhibitors [in which greater than 95% knockdown in the heart for at least 2 months was achieved (Bernardo et al., 2012b, 2014a, 2016b)]. It is also worth noting that others have reported a modest decrease in miRNA expression (∼40–60% decrease) in the heart following administration of TuDs (both using a U6 promoter and AAV9 serotype) (Jeong et al., 2018; Nie et al., 2018) in comparison to LNA inhibitors.

A limitation of our study is that we have not tested the half-life of our constructs in the heart, but it is expected to be long lasting. Previous studies using AAV vectors have demonstrated vector sequences were present in cardiac tissues from patients for at least 31 months (Zsebo et al., 2014). Further, our previous studies using the same AAV6-CMV cassette to deliver mRNAs demonstrated that the AAV is detected in the heart at 8 weeks (Weeks et al., 2012; Bernardo et al., 2018).

## Future Developments for miRna-Targeting Therapeutics – New Technologies

There is intense effort to identify agents that are capable of targeted delivery of oligonucleotides to tissues and cells. Both viral and non-viral options, as well as web-based tools for *in silico* testing are currently being developed and are discussed below.

### Choice of AAV Serotype and Promoter

Recombinant vectors have been used for miRNA loss and gain of function research, as it allows for greater flexibility (choice of AAV serotypes, promoters and reporter genes), and achieves persistent transgene expression conferring long duration of mRNA suppression (Bass-Stringer et al., 2018). In our study, we used AAV6 with the CMV promoter, as we consistently observe this to be cardiac selective in our hands (Gregorevic et al., 2004; Weeks et al., 2012; Prakoso et al., 2017; Bernardo et al., 2018). However, neither the sponge nor TuD approach inhibited miR-34 in the heart. It is noteworthy that the use of AAV6 with a CMV promoter was sufficient to downregulate miR-206 by direct intramuscular injection in skeletal muscle (Winbanks et al., 2013). In several different human cell lines, it was demonstrated that the choice of promoter used to drive RNAi expression was of critical importance to allow effective mRNA target knockdown; the U6 promoter provided knockdown of >90% in all cell lines tested whereas the CMV promoter provided knockdown of 50–92% depending on the cell line (Lebbink et al., 2011). Thus, it is possible that the CMV promoter together with AAV6 is not sufficiently potent to drive expression of the tough decoy and sponge approaches in the heart *in vivo* via systemic delivery. The use of specific promoters, such as α-myosin heavy chain, whilst increasing targeted delivery to the heart, may elicit a decrease in the transcription level, affecting efficiency (Raso and Dirkx, 2017). The first successful demonstration of using the TuD method in the heart was reported in 2018. The cardiotropic AAV9 serotype was used with the U6 promoter (Jeong et al., 2018). Whilst this achieved ∼60% knockdown of the miRNA of interest in the heart (Jeong et al., 2018), the U6 promoter is active across different cell and tissues types, and the effect of this tough decoy on other tissues was not reported (Jeong et al., 2018). Further, there have been reports of toxicity associated with the use of U6 promoter in animal studies (Grimm et al., 2006; Giering et al., 2008; McBride et al., 2008; Borel et al., 2014). Methods to improve cardiac tropism include adopting an AAV pseudotyping strategy (Muller et al., 2006), or optimizing the promoter of choice to specifically target cell types of interest (Piras et al., 2013), although these have yet to be employed to miRNA targeted therapy.

### Microbubbles

A novel theranostic approach for targeted miRNA delivery has been described using ultrasound microbubbles as carriers, allowing both imaging and targeted therapy of miRNA mimetics in abdominal aortic aneurysm (AAA) (Wang et al., 2018). Microbubbles were coupled to a single chain antibody specifically targeting inflamed endothelial cells, and ultrasonic bursting provided additional selectively for transfection of miRNA mimetics (Wang et al., 2018). This approach may pave the way for the technology to be used to enhance tissue specificity of miRNA inhibitors, although antibodies that specifically target cardiomyocytes need to be developed.

### Nanoparticles/Liposomes

Nanotechnology represents a promising approach for efficient delivery of therapeutics. These can be injected intravenously, circulate in the body for a long period of time, and can be targeted to specific organs or cell types by adding cell-surface receptor ligands to bind to desired tissues. Nanoparticles have been extensively used in the cancer field to deliver therapeutic miRNAs (Babar et al., 2012; Campani et al., 2016), but is an emerging technology in the heart field. Di Mauro et al. (2016) generated negatively charged calcium phosphate nanoparticles (CaP-NPs) for the delivery of miRNAs to cardiac cells *in vitro* and *in vivo*, although further studies are required to evaluate long-term *in vivo* toxicity (Di Mauro et al., 2016). In a subsequent study by the same group, inhalation therapy of CaP-NPs was effective for delivering nanoparticle-based therapeutic peptides to the diseased heart (Miragoli et al., 2018). Dvir et al. (2011) conjugated liposomes with a ligand specific to the angiotensin II type 1 receptor, and when administered intravenously, the nanoparticles were able to specifically target the infarcted heart, demonstrating that this approach could reduce toxicity of delivered drugs and increase local therapeutic effect (Dvir et al., 2011). Although these studies are not limited to miRNA inhibitors, it opens up new avenues of investigation for therapeutic targeting of miRNAs. Coating nanoparticles with targeting ligands may improve selective uptake, and nanoparticles can be coated with bioactive polymers (e.g., polyethylene glycol) to increase circulation time when administered systemically. Through such modifications, nanoparticles can be engineered to overcome physiological or cellular delivery barriers.

### miRNAsong Tool

A new user-friendly, freely available web-based software tool has been developed that allows for generation and *in silico* testing of miRNA sponge constructs (Barta et al., 2016). The tool generates miRNA sponge constructs for specific miRNAs and miRNA families or clusters, and tests them for potential binding to miRNAs in selected organisms. This is an important property, as sponge sequences have to be carefully designed to avoid binding to other miRNAs causing off-target effects and possible false-positive results (Barta et al., 2016).

### WPRE-Linked Tough Decoy miRNA Sponges

To improve the miRNA suppression potential of the TuD hairpin, Hollensen et al. (2017) inserted an RNA element, (Woodchuck hepatitis virus [WHV] post-transcriptional regulatory element [PRE], WPRE) upstream of the TuD hairpin. The WPRE RNA element has been shown to enhance gene expression *in vitro* and *in vivo*. When inserted upstream of the TuD, the WPRE facilitated a significant increase in the miRNA suppression activity of the TuDs, thus providing new guidelines for the design and production of optimized miRNA sponges (Hollensen et al., 2017).

### Biomaterials

A more recent study developed a biomaterial-based miRNA delivery system to target cardiomyocyte proliferation after cardiac injury. Specifically, Wang et al. (2017) developed an injectable hyaluronic acid hydrogel for the local and sustained delivery of miRNA mimics to the heart and showed that a single injection of this gel in the mouse heart led to increased cardiomyocyte proliferation and improved cardiac function after MI (Wang et al., 2017).

## Conclusion

The discovery of short non-coding miRNAs, and our increasing understanding of their functions, has presented potential therapeutic applications for cardiovascular disease. A number of studies using miRNA inhibitors with different chemical modifications have shown promise in preclinical models of HF. Although some studies demonstrate the efficacy of miRNA sponges and TuDs in inhibiting miRNA activity, there is still a need for a more efficient, safe and cardiac-specific drug delivery system. New technologies are emerging, including the use of microbubbles and bio-engineered nanoparticles, which may make a cardiac-specific miRNA drug for patients with HF a possibility.

## Methods

### Experimental Animals

Animal care and experimentation were conducted in accordance with the Australian Code for the Care and Use of Animals for Scientific Purposes (National Health and Medical Research Council of Australia, 8th edition, 2013), and approved by the Alfred Medical Research and Education Precinct (AMREP) Animal Ethics Committee. Male 10 week old mice, either on a C57BL/6 or FVB/N background, were used in the present study. Six mice on a C57BL/6 background were used in the TuD-miR-34a study (three mice received AAV6-control vector; three mice received AAV6-TuD-miR-34a). Twelve mice on a FVB/N background were used in the miR-34-sponge study (three mice received AAV6-control 15mer sponge, three mice received AAV6-miR-34-15mer sponge; three mice received AAV6-control 8mer sponge, three mice received AAV6-miR-34-8mer sponge). Each mouse was administered 1 × 10^12^ vector genomes of control, TuD or sponge intravenously as previously described (Bernardo et al., 2018). Mice were housed in a 12 h light-dark cycle and had access to food *ad libitum*. After 8 weeks, tissue was harvested for molecular analysis.

### Generation of Sponges and a Tough Decoy

The TuD-miR-34a sequence, miR-34 sponge sequences and miR control sponge sequences was modified by adding a *Not1* restriction site to the 5′ end and a *HindIII* restriction site to the 3′ end. The constructs were made by GenScript (Jiangsu Province, China) and cloned into an AAV6 plasmid using standard cloning techniques. The plasmid contained a CMV promoter and a synthetic poly(A). The AAV6-TuD-miR-34a, AAV6-control, and AAV6-miR-34 sponge and control vectors were produced as previously described (Weeks et al., 2012; Bernardo et al., 2018).

### Cell Culture

H9c2 cells (CellBank Australia, Westmead, NSW, Australia) were seeded at 100,000 cells/well (passage 13). H9c2 cells (25 mM Dulbecco’s Modified Eagle Medium, 10% fetal bovine serum, and 1% Penicillin Streptomycin) were transfected with 4 μg of AAV6-CMV-control or AAV6-CMV-miR-34 15mer sponges plasmid DNA in 6-well plates using Lipofectamine 2000 (Life Technologies). After 72 h, RNA was isolated and gene expression of miR-34 family members was assessed by quantitative PCR (qPCR).

### RNA Isolation

Total RNA was isolated from frozen mouse ventricles or H9c2 cells using TRI reagent (Sigma-Aldrich, St. Louis, MO, United States) and quantified on a Nanodrop^TM^ Spectrometer (Thermo Fisher Scientific, Waltham, MA, United States).

### DNA Extraction

Genomic DNA was isolated from frozen mouse ventricles using a lysis buffer (100 mM Tris–HCl, pH 8.5, 5 mM EDTA, 200 mM NaCl, 0.2% SDS and 0.1 mg/ml of proteinase K) and incubated at 55°C overnight for enzymatic digestion of proteins and non-nucleic acid cellular components. DNA was then purified using a mixture of phenol:chloroform:isoamyl alcohol (25:24:1) which promotes the partitioning of lipids and cellular debris into the organic phase, leaving isolated DNA in the aqueous phase. Following centrifugation, the aqueous phase containing the purified DNA was concentrated by ethanol precipitation and quantified on a Nanodrop^TM^ Spectrometer (Thermo Fisher Scientific).

### Quantitative PCR (qPCR)

For qPCR analysis of miRNAs, 50 ng of total RNA was reversed transcribed using the Applied Biosystems^®^ TaqMan^®^ MicroRNA Reverse Transcription Kit (Life Technologies, Carlsbad, CA, United States) in accordance with the manufacturer’s instructions. To detect the level of miR-34a, miR-34b, and miR-34c, qPCR was performed using TaqMan MicroRNA Assays (Life Technologies) on an Applied Biosystems Quant Studio 6 and 7 real-time PCR instrument. Expression was normalized against snoU6 using the 2^−ΔΔCT^ method of quantification. For mRNA expression analysis, 1 μg of total RNA was reverse transcribed using the High Capacity RNA-to-cDNA kit (Life Technologies) according to manufacturer’s recommendations. qPCR was performed using TaqMan^®^ probes (Life Technologies) and amplified on an Applied Biosystems 7500 real-time PCR instrument according to manufacturer’s instructions. Hypoxanthine phosphoribosyltransferase 1 (*Hprt1*) was used to standardize for cDNA concentration and data was analyzed using the 2^−ΔΔCT^ method of quantification. Vector genomes incorporation was determined by RT-qPCR using primers specific to the CMV promoter [forward, 5′-gcggtaggcgtgtacggtgg-3′; reverse,5′-cgtggatggcgtctccaggc-3′ (Weeks et al., 2012; Bernardo et al., 2018)], 100 ng of DNA, and amplified on an Applied Biosystems 7500 real-time PCR instrument.

### Statistics

Statistical analyses were performed using GraphPad Prism (Version 7, La Jolla, CA, United States). Results are presented as mean ± SEM. Differences between groups were identified using an unpaired *t*-test or One way ANOVA with Fisher’s *post hoc* test. A value of *P* < 0.05 was considered significant. All relative units are expressed as a fold change with the relevant control group normalized to 1.

## Author Contributions

JM and BB conceived the study and designed the experiments. BB wrote the manuscript, designed the miRNA sponges and tough decoys, performed the experiments, and analyzed the data. PG contributed to the development of miRNA sponges and tough decoys. RR and JM critically reviewed and edited the manuscript. All authors approved the final version of the manuscript to be published.

## Conflict of Interest Statement

The authors declare that the research was conducted in the absence of any commercial or financial relationships that could be construed as a potential conflict of interest.
